# Breaking barriers: centering researchers with lived experience in psychiatric neuroscience

**DOI:** 10.1038/s44277-025-00048-7

**Published:** 2025-11-13

**Authors:** Uma R. Chatterjee, Maya C. Schumer, Devin P. Effinger, Nev Jones, Noel A. Vest, Michael E. Cahill, Brandon K. Staglin, Eric J. Nestler

**Affiliations:** 1 University of Wisconsin School of Medicine and Public Health, 750 Highland Ave, Madison, WI 53726 USA; 2 Neuroscience Training Program, University of Wisconsin-Madison, 1111 Highland Ave 9533 WIMR II, Madison, WI 53705 USA; 3 Psychotic Disorders Division, McLean Hospital, 115 Mill St, Belmont, MA 02478 USA; 4 Department of Psychiatry, Harvard Medical School, 22 Shattuck St, Boston, MA 02115 USA; 5 Department of Psychiatry, University of Colorado Anschutz Medical Campus, 12700 East 19th Ave, Aurora, CO 80045 USA; 6 School of Social Work, University of Pittsburgh, Cathedral of Learning 2117, 4200 Fifth Ave, Pittsburgh, PA 15260 USA; 7 Department of Community and Health Sciences, Boston University School of Public Health, 801 Massachusetts Ave, Boston, MA 02118 USA; 8 Department of Comparative Biosciences, University of Wisconsin-Madison, 2015 Linden Drive, Madison, WI 53706 USA; 9 One Mind, 1570 Bella Oaks Lane, Rutherford, CA 94573 USA; 10 Nash Family Department of Neuroscience, Friedman Brain Institute, Icahn School of Medicine at Mount Sinai, 1 Gustave L Levy Place, New York, NY 10029 USA

**Keywords:** Careers, Bipolar disorder, Schizophrenia, Addiction, Translational research

## Abstract

Researchers with lived experience (RWLE) of serious mental illness or substance use disorders (SMI/SUD) bring critical dual expertise to psychiatric neuroscience as both scientists and individuals directly affected by the conditions they study. Yet their participation and leadership remain profoundly limited by entrenched stigma, disclosure risks that can obstruct promising career trajectories, lack of mentorship from senior RWLE, and the absence of structural protections against discrimination and exclusion. These systemic barriers silence voices that can help transform the field’s understanding of mental illness and its biological underpinnings. Drawing on the authors’ lived and/or professional experiences, this Perspective challenges the assumption that lived experience introduces bias, reframing it as a source of empirical strength, innovation, and epistemic diversity. Here, the authors propose structural reforms to reshape admissions, mentorship, and leadership pathways. Centering RWLE is both a scientific necessity and an ethical imperative for advancing a more equitable and representative psychiatric neuroscience.

## Lay Summary

Researchers who live with serious mental illness or substance use disorders bring unique insight to psychiatric neuroscience, yet they remain underrepresented in the field. This paper calls for recognizing and removing the barriers that limit their participation and leadership. Including these researchers strengthens the science, improves the relevance of the research to real-world needs, and helps to ensure that research about mental illness includes those who live it.

## Why this paper, why now

Researchers with lived experience (RWLE) of serious mental illness (SMI) or substance use disorders (SUD) bring a dual expertise to their field: scientific training grounded in an empirical understanding of the conditions they experience combined with expertise rooted in experiential knowledge. Yet, in psychiatric neuroscience–a field tasked with uncovering the brain mechanisms of mental illness and addiction–RWLE remain largely absent from leadership and mostly invisible in authorship and academic culture.

Despite comprising more than one in four adults in the United States (28.7%) [1], people with disabilities represent only about 3% of the STEM workforce [2]. This stark disparity underscores the systemic absence of disabled scholars from the sciences [3]. Within this already underrepresented population, those with psychiatric disabilities and even more pointedly the highly stigmatized diagnoses typically associated with SMI are even less visible, reflecting compounded stigma and structural barriers that uniquely marginalize RWLE in academic neuroscience. Considering these barriers, it is thus important to acknowledge that these statistics likely do not reflect the true number of RWLE in the STEM workforce.

This paper emerges from some of the authors’ experiences as psychiatric and neuroscience researchers living with SMI/SUD and as leading advocates driving systemic change toward centering lived experience. This is the first effort of its kind within these fields and a call for structural reform. The authors collectively represent diverse diagnoses, research methods spanning preclinical, clinical, and translational neuroscience, intervention research, and multiple career stages. Importantly, multiple co-authors openly live with SMI/SUD. Other authors, although not living with SMI themselves, contribute their insights as principal investigators whose mentorship of RWLE trainees and collaboration with colleagues has informed their recognition of the unique value such inclusion brings to psychiatric neuroscience. Collectively, the group spans a wide range of research approaches, including cellular and molecular neuroscience, biochemistry, systems and behavioral neuroscience, clinical psychiatry, human neuroimaging, and public mental health. The authors include researchers at every career level: one PhD student, two postdoctoral fellows, one assistant professor, two associate professors, one full professor, and the co-founder and lead officer of a global research nonprofit organization. The authors also reflect varied educational pathways, from traditional to non-traditional, including those disrupted by incarceration, intensive treatment and termination from academic programs.

Building on prior work documenting the stigma, discrimination, and disclosure risks facing RWLE in mental health services and intervention research [1, 4–6], this paper highlights how these challenges also manifest within psychiatric neuroscience. The aim is twofold: first, to articulate the systemic barriers facing RWLE across career stages; second, to reframe LE as an epistemic and scientific strength rather than a liability.

## Barriers and stigma in psychiatric neuroscience

Despite a growing emphasis on inclusiveness in research and academia overall, psychiatric neuroscience remains shaped by long-standing structural barriers for people with a range of disabilities and a narrow conception of objectivity that treats researcher identity as bias [5]. Disclosure of LE–whether in graduate applications, grant proposals, or job searches–has historically been discouraged, with trainees warned it will be held against them [6, 7], and even often referred to as a “kiss of death” in graduate admissions [8].

Within biological sciences broadly, ableism is well-documented [9], but psychiatric neuroscience adds another layer: the pervasive stigma surrounding SMI/SUD [10]. Unlike cancer researchers who may openly identify as survivors without assumptions about their competence, RWLE in psychiatric neuroscience confront stereotypes casting them as “broken,” “unreliable,” or “too close” to the topic for scientific rigor. Ironically, the very symptoms for which the field compassionately works towards finding solutions are used to question RWLE’s capabilities, collapsing illness and identity into professional liability. Moreover, across the spectrum of psychiatric diagnoses, even as public attitudes improve in some cases (e.g., depression), stigma has in fact steadily worsened for schizophrenia and alcoholism, with the majority of Americans surveyed expressing unwillingness to work closely with someone with either condition as of 2018 [11].

Even allies in adjacent disciplines often struggle to recognize the relevance and value of RWLE in psychiatric neuroscience, reflecting a broader marginalization of experiential knowledge in the “psy-fields” [5]. NIH reviewers have penalized applicants both for disclosure and nondisclosure, reflecting inconsistent standards driven more by stigma than by evidence [6, 12]. As a result, RWLE face a paradox: disclosure is deemed risky, yet omission is penalized, perpetuating silence and exclusion.

## Career stages: distinct challenges across the pipeline

Barriers facing RWLE shift across career stages but share common roots in ableism, disclosure, and stigma, compounded by the imposition of the same frequently-unmeasurable productivity norms (i.e., “publish or perish” [13]) for all researchers, despite the additional structural and health-related obstacles RWLE must navigate. Importantly, these obstacles are further compounded by intersectional stigma among researchers who also hold gender, non-psychiatric disability and racial minority identities and/or who have experienced significant socioeconomic disadvantage.

## Early Career

From undergraduate labs to graduate admissions and postdoctoral fellowships, junior RWLE frequently encounter the “status quo” expectation of nondisclosure. Applicants with training gaps or medical leaves face pressure to explain their records while knowing any admission of psychiatric disability may trigger bias or discrimination [6]. In some cases, some RWLE are effectively forced into disclosure when psychiatric crises have intersected with law enforcement or resulted in arrest records, as such histories often surface in background checks or institutional screenings–further compounding stigma and bias long after recovery or stability has been achieved. Such disclosures often carry the perception of being a “risk” or “red flag,” which discourages and punishes openness even when explanations are warranted [7]. Unlike many physical illnesses, the fluctuating and often invisible nature of SMI/SUD–and the different accommodations they may require–frequently forces disclosure during crises, leaving trainees vulnerable to discrimination precisely when support is most needed. The consequences at this stage can be especially severe: disclosure can result in lost admissions offers, rescinded opportunities, or withheld mentorship; effectively excluding many RWLE from the field before their scientific careers can take hold. In this way, promising psychiatric neuroscience careers are often cut short at the outset, not because of ability or potential, but because of entrenched stigma and a lack of understanding by some PIs and their institutions on how to support such trainees.

Moreover, the pressure to appear “high-functioning” pervades early-career experiences: RWLE often feel compelled to overperform to counter stigma not only from their peers and colleagues but also the impact of stigmatizing language in research papers, only for their productivity output to be penalized when symptoms flare or accommodations are requested. This catch-22 can perpetuate internalized stigma and foster cycles of burnout, masking, self-silencing, and relentless pressure to push themselves in ways that may be incongruent with their health needs, sometimes to the point of symptom exacerbation or relapse. Stigmatizing language in research environments (e.g., terms like “schizophrenic”, “addict”, or “abnormal”) compounds these harms, reinforcing the biases that RWLE encounter daily and pathologizing, rather than valuing, their identities. Professional societies, journals, and conference organizers should adopt and enforce language policies that explicitly discourage stigmatizing terminology and require mindful, person-centered descriptions of psychiatric conditions.

## Mid-career

For RWLE establishing independent labs, disclosure becomes a double-edged sword. Some, like the authors, report mentorship and funding opportunities emerging from openness–particularly when senior allies value LE as complementary subject matter expertise. Others encounter sensationalization, where living with SMI/SUD is reframed as a “superpower” rather than recognized in its complexity, or experience discrimination and exclusion from leadership despite strong research records [5].

At this career stage, mid-career RWLE are often newly responsible for leading research groups and mentoring junior scientists, which places their professional authority under heightened scrutiny. Disclosure risks may shift how trainees and colleagues perceive their competence, potentially undermining the credibility needed to attract talented team members, secure collaborations, and obtain funding–all of which are needed to remain in the field. As is true for other marginalized faculty, publicly-disclosed RWLE, a tiny minority of researchers at the associate level and above, can become magnets for undergraduates and junior trainees with LE from across the country seeking favorable mentorship and support, while creating a high burden of emotional labor and mentoring commitments beyond what can be feasibly managed. In addition, for RWLE who employ entire labs of junior staff and trainees with LE, myriad challenges can arise, including handling multiple disability leaves or juggling identical expectations to non-LE labs while attempting to accommodate a high proportion of trainees with disabilities. Some mid-career RWLE may calculate that maintaining silence is safer for preserving professional boundaries required to sustain their labs.

For those actively engaged in mentoring mid-career and beyond, a further source of significant emotional labor involves coaching junior trainees through the difficult decisions involved in disclosure and the harms that can stem from early inclusion in research projects in tokenistic, exploitative, or extractive ways [14].

## Senior career

For the rare publicly-disclosing RWLE in senior positions, including full professors and primary investigators of large extramurally funded centers or teams, additional burdens emerge: still more heightened mentorship responsibilities, the navigation of disclosure politics in leadership roles, and challenges of institutional inertia resistant to systemic reform [5]. Their visibility, while groundbreaking, often comes without structural protections or pipelines to ensure that future generations face fewer barriers. Limited senior RWLE visibility often arises not only from exclusion itself but from fears that disclosure will further restrict leadership opportunities.

Mentorship gaps further compound these barriers. Few publicly-disclosing senior RWLE exist in psychiatric neuroscience, and those present often conceal their experiences to avoid stigma. This lack of representation denies trainee and early-mid career RWLE visible role models and perpetuates the false perception that RWLE cannot succeed in the field. The invisibility of those who came before risks reinforcing structural exclusion at every stage.

## Reframing ‘bias’ as strength and knowledge

There remains a common assumption in science that LE may introduce bias and pose challenges to objectivity [15, 16]. The authors challenge this framing. In practice, RWLE are held to the same standards of scientific excellence as their peers; their methodological rigor, statistical competence, and experimental design skills are no different. What differs is the integration of experiential knowledge–offering insights that can be inaccessible to researchers without LE, including insights into phenomenology [17, 18] and/or etiology [19, 20] that explicitly challenge the status quo. Indeed, we argue that, in many cases, LE decreases susceptibility to bias and conflicts of interest. RWLE frequently enter the field motivated by a deep sense of purpose and a life calling to advance understanding of and treatments for conditions they themselves have experienced [21]. In this sense, the drive to find genuine solutions may in fact function as a safeguard against the kinds of overinterpretation and premature claims that can mislead the field and slow therapeutic and translational progress. Although some in the field view strong personal motivation as a liability that could influence research questions or interpretations, we contend that, when paired with methodological rigor, ethical integrity, and reflexivity, LE strengthens–not weakens–the integrity and relevance of the science.

Rather than undermining neuroscience, RWLE expand and evolve it. Their perspectives challenge dominant and overly reductionistic models equating SMI solely with pathology [22], bringing nuance to constructs like “function,” “impairment,” “stability,” and “recovery” that are often defined without input from those living the realities under study [22]. Far from introducing subjectivity, the contributions of RWLE strengthen psychiatric and biological research validity, expand conceptual frameworks, and highlight blind spots in prevailing theories, animal models, imaging paradigms, and clinical metrics, ensuring that research questions reflect lived realities rather than solely outsider assumptions [6, 23–27], and accelerates translation from bench to bedside [4, 5, 28]. However, to the authors’ knowledge, there are few explicit contributions from visible RWLE in the neurosciences. By integrating multi-level scientific, experiential, and critical knowledge informed by diverse identities and contexts, RWLE advance a pluralistic approach to psychiatric neuroscience–one that moves beyond binary notions of “normal” vs. “abnormal” toward models that capture the full complexity and heterogeneity of psychiatric disorders [29].

This also raises questions of *epistemic exclusion*–a form of discrimination that occurs when marginalized groups are not given credit as “epistemic agents” and are instead excluded from the process of creating knowledge and meaning. In mental health research, epistemic exclusion has historically devalued the expertise of those with LE in favor of clinical or professional authority, often leading to their perspectives being interpreted or defined by outsiders [30, 31]. As members of the scientific community, RWLE inherently embody epistemic diversity. Neuroscience has long recognized the value of interdisciplinary teams [32]; integrating experiential knowledge follows the same logic. As the authors argue, this dual expertise in science and LE is additive, not adversarial, strengthening validity, translational relevance, ethical integrity, and epistemic justice across the research pipeline.

RWLE have collectively contributed to the psychiatric and neuroscience literature on their conditions; some of the authors, who are RWLE, have both identified critical knowledge gaps and generated perspectives that have meaningfully advanced these fields. URC (LE with obsessive-compulsive disorder[OCD]) identifies a major translational gap in current animal models of OCD pathology, which predominantly capture nonspecific repetitive behaviors rather than compulsive behaviors driven by obsessions–intrusive, unwanted, and distressing thoughts–and is developing novel and modified behavioral assays with MEC to more accurately model the obsessional dimension and its associated safety and compulsive responses [33]. DPE (LE of SUD) assisted in developing a non-human primate economic choice model incorporating naturalistic rewards to better capture real-world decision-making in SUD [34]. His LE with medication-assisted treatment later proved essential in interpreting why methadone pretreatment failed to shift choice away from opioids–illuminating that such drugs alleviate withdrawal but do not eliminate desire. MCS (LE of bipolar disorder[BD]), through large-scale coordinate-based meta-analyses of BD neuroimaging studies [35], cross-sectional independent replication [36], and network-level approaches, expanded our conceptualization of the underlying functional neurobiology of BD, shifting beyond emotion-limbic models to reflect the complexity of BD phenomenology across risk, resilience, and established illness. NJ (LE of schizophrenia) has empirically challenged conventional understanding of the phenomenology of psychosis, including important work challenging our understanding of ‘voices’ as fundamentally auditory [17, 37]. NAV (LE of SUD and prior work as a SUD counselor) drew on his clinical insights to identify distinct patient subgroups based on co-occurring patterns of depression and substance use among patients in treatment for opioid use disorder [38, 39]. These are just several illustrative examples of how RWLE in psychiatric neuroscience are reconceptualizing and expanding our understanding of SMI/SUD. Importantly, by integrating their dual perspectives and advocating for their communities, RWLE also help to destigmatize both the science and research practices surrounding their conditions.

## Looking ahead: building the pipeline, changing the field

The field of psychiatric neuroscience would greatly benefit from actively recruiting and supporting RWLE: such inclusion would broaden the range of expertise and perspectives available to uncover novel mechanisms and develop more effective treatments. The authors propose five priorities:

1. **Reform application, review and recruitment processes**. Graduate admissions, grant panels, and faculty hiring committees require training to prevent disclosure-related discrimination [6]. Granting agencies, both public and private, should explicitly identify, support, and sponsor RWLE leadership on projects that study their communities [5].
2. **Develop funding mechanisms, accessible training pathways, and mentorship networks for RWLE**. Funding mechanisms, including fellowships and career development programs, that support RWLE at all career stages–undergraduate through faculty–would build infrastructure and accessible pipelines for the sustained inclusion of these individuals in psychiatric neuroscience [5, 26]. RWLE facing career barriers related to their condition were formerly eligible for NIH diversity mechanisms (e.g., diversity supplements, Fs, Ks); their termination has left a continued gap in support. Partnerships between professional societies and the nonprofit sector represent one vital path to provide such support.
3. **Expand RWLE professional development and representation**. Workshops, panels, and keynote invitations should treat RWLE as scientific experts, not diversity tokens, integrating their unique perspectives across mainstream programming rather than siloed sessions. This also means creating professional pipelines, leadership opportunities, and sustained roles in shaping research agendas, editorial boards, and professional societies. Providing RWLE with the same visibility and authority as other scholars not only strengthens science by grounding it in LE, but also signals to emerging investigators that their perspectives are valued and vital.
4. **Promote epistemic inclusivity and embrace pluralism**. Experiential knowledge must be recognized as a form of expertise, not simply “anecdote” or “storytelling” [22]. This shift requires moving well beyond deficit-based models framing RWLE solely through stigma or pathologized as “patients” and instead valuing their insight and contributions to theory, methods, and interpretation, including reconceptualizing neurobiological models of SMI/SUD to reflect the range and complexities of LE. The authors invoke the principle of “nothing about us without us,” long established in promoting the rights of people with disabilities [40], to argue that science about psychiatric disorders must be co-created with those with LE.
5. **Foster leadership and power-sharing**. As stated by Glenn E. Martin, “people closest to the problem are closest to the solution, but furthest from the power and resources” [41]. Inclusion cannot end with participation; RWLE belong in principal investigator roles, senior authorship positions, editorial boards, study sections, and academic leadership and administrative roles. Fairness and equal opportunity demand moving beyond tokenism toward genuine shared decision-making power and collaborative leadership models.

## Conclusion

RWLE are already actively and uniquely contributing across the psychiatric neuroscience workforce, even when their presence goes mostly unrecognized. Many weigh disclosure risks amid pervasive stigma against career survival in a system that simultaneously demands and penalizes openness. Those who speak out often do so without structural protections, carving paths for others while confronting barriers alone. The limited visible representation of RWLE–particularly in senior roles–reflects both their systematic exclusion from leadership and the many who remain undisclosed due to historical stigma, discrimination, and lack of safety. Importantly, choosing not to disclose is not a shortcoming but a personal and nuanced decision to be respected; recognition, inclusion, and opportunity should never depend on disclosure. RWLE deserve the ability to work openly as their full selves, sharing both their scientific expertise and LE perspectives.

Including RWLE is not charity, identity politics, or public relations, nor should it be a “checked box” or afterthought when designing studies. It is both an ethical obligation and a scientific imperative. LE strengthens research validity, expands conceptual frameworks, and accelerates translation from bench to bedside. A future where RWLE are fully valued and recognized for their unique contributions, hold equitable leadership, shape research agendas at all stages [28], and train the next generation will produce not only better psychiatric neuroscience but fairer systems for those within and beyond the academy.

The goal is simple: to augment the field such that psychiatric neuroscience benefits from the full breadth of human expertise–including those who live the realities it seeks to understand.

### Citation diversity statement

The authors have attested that they made efforts to be mindful of diversity in selecting the citations used in this article.
